# Supplementary material for: Serological Surveillance of COVID-19 Hospitalized Patients in Réunion Island (France) Revealed that Specific Immunoglobulin G Are Rapidly Vanishing in Severe Cases
Source: J Clin Med. 2020 Nov 27;9(12):3847. doi: 10.3390/jcm9123847 (PMC7761058; doi:10.3390/jcm9123847)
Supplement: Supplementary file 1 [file jcm-09-03847-s001.pdf]

Supplementary Materials

Table S1. Baseline characteristics of symptomatic COVID-19 ICU and non-ICU patients hospitalized at CHU of La Réunion. Patients were hospitalized for at least two weeks. Among the 26 eligible patients, 12 were admitted in ICU.

	**Total (N = 26)**		**Non-ICU (N = 14)**		**ICU (N = 12)**		***P*-value**
Age (years), mean [SD]	64 [16]		67 [17]		61 [15]		0.33
Hospital stay (days), median [IQR]	22 [18-29]		20 [18-26]		28 [17-41]		0.16
Day of admission in ICU (after symptom onset), mean [SD]	9 [5]				9 [5]		
**Sex**	N	%	N	%	N	%	
Female	5	19	4	29	1	8	0.33
Male	21	81	10	71	11	92	0.33
**Oxygen therapy**	N	%	N	%	N	%	
Maximal oxygen flow received (⩽ 5 Liters/minute)	16	62	14	100	2	17	**< 0.0001**
Maximal oxygen flow received (> 5 Liters/minute)	10	38	0	0	10	83	**< 0.0001**
**Comorbidities**	N	%	N	%	N	%	
Diabetes	6	23	1	7	5	42	0.07
Hypertension	13	50	6	43	7	58	0.69
Obesity	5	19	3	21	2	17	1.0
Dyslipidemia	7	27	4	29	3	25	1.0
Asthma	3	12	2	14	1	8	1.0
Cardiopathy	4	15	2	14	2	17	1.0
Malignancy	2	8	0	0	2	17	0.20
Renal insufficiency	5	19	1	7	4	33	0.14
Total	45	100	19	42	26	58	0.20

ICU: intensive care unit; IQR: interquartile range; SD: standard deviation.

Table S2. Laboratory findings of symptomatic COVID-19 ICU and non-ICU patients hospitalized at CHU of La Réunion. Patients were hospitalized for at least two weeks. Among the 26 eligible patients, 12 were admitted in ICU.

	Median [IQR] or mean [SD] [a]				
	**Normal range**	**Total (N = 26)**	**Non-ICU (N = 14)**	**ICU (N = 12)**	***P*-Value**
($\times 10^9$/L)					
White blood cell count	4.0-10.0	13.8 [8.1-19.9]	10.3 [6.5-14.6]	17.4 [12.5-22.9]	**0.02**
Neutrophil count	1.5-7.0	10.9 [6.0-17.2]	7.9 [4.1-11.2]	14.1 [9.7-20.1]	**0.03**
Lymphocyte count	1.5-4.0	2.7 [1.7-3.6]	2.3 [1.7-3.3]	3.1 [1.7-4.2]	0.34
Monocyte count	0.2-1.0	1.2 [0.8-1.6]	0.9 [0.6-1.2]	1.6 [1.0-1.8]	**0.03**
Platelet count	150-400	415 [164]	431 [165]	397 [169]	0.61
(U/L)					
LDH	125-220	374 [112]	321 [87]	436 [108]	**0.009**
ALP	40-150	137 [85-201]	110 [72-241]	147 [107-164]	0.66
GGT	12-64	180 [79-268]	134 [44-260]	219 [112-269]	0.27
ALT	5-55	97 [32-122]	59 [28-116]	114 [38-143]	0.29
AST	6-40	62 [42-100]	61 [42-112]	66 [42-96]	0.84
(µmol/L)					
Total bilirubin	1.71-20.5	14.0 [9.0-19.5]	14.0 [9.0-17.5]	14.0 [9.3-25.3]	0.68
Creatinine	64-104	99 [73-113]	99 [73-108]	92 [69-135]	0.96
(ng/L)					
D-dimers [b]	0-500	1309 [549-3647]	849 [504-1940]	4698 [1775-10285]	**0.03**
(g/dL)					
Hemoglobin	13.0-18.0	10.8 [2.4]	11.7 [1.9]	9.7 [2.5]	**0.04**
(mg/L)					
CRP	0-5	135 [94]	108 [71]	166 [111]	0.12

ALP: alkaline phosphatase; ALT: alanine aminotransferase; AST: aspartate aminotransferase; CRP: C-reactive protein; GGT: gamma-glutamyl transferase; LDH: lactate dehydrogenase. [a]Median and IQR were used for data that are not normally distributed and mean and SD were used for data that are normally distributed. [b]For D-dimers, data were available for 9 non-ICU and 4 ICU patients.

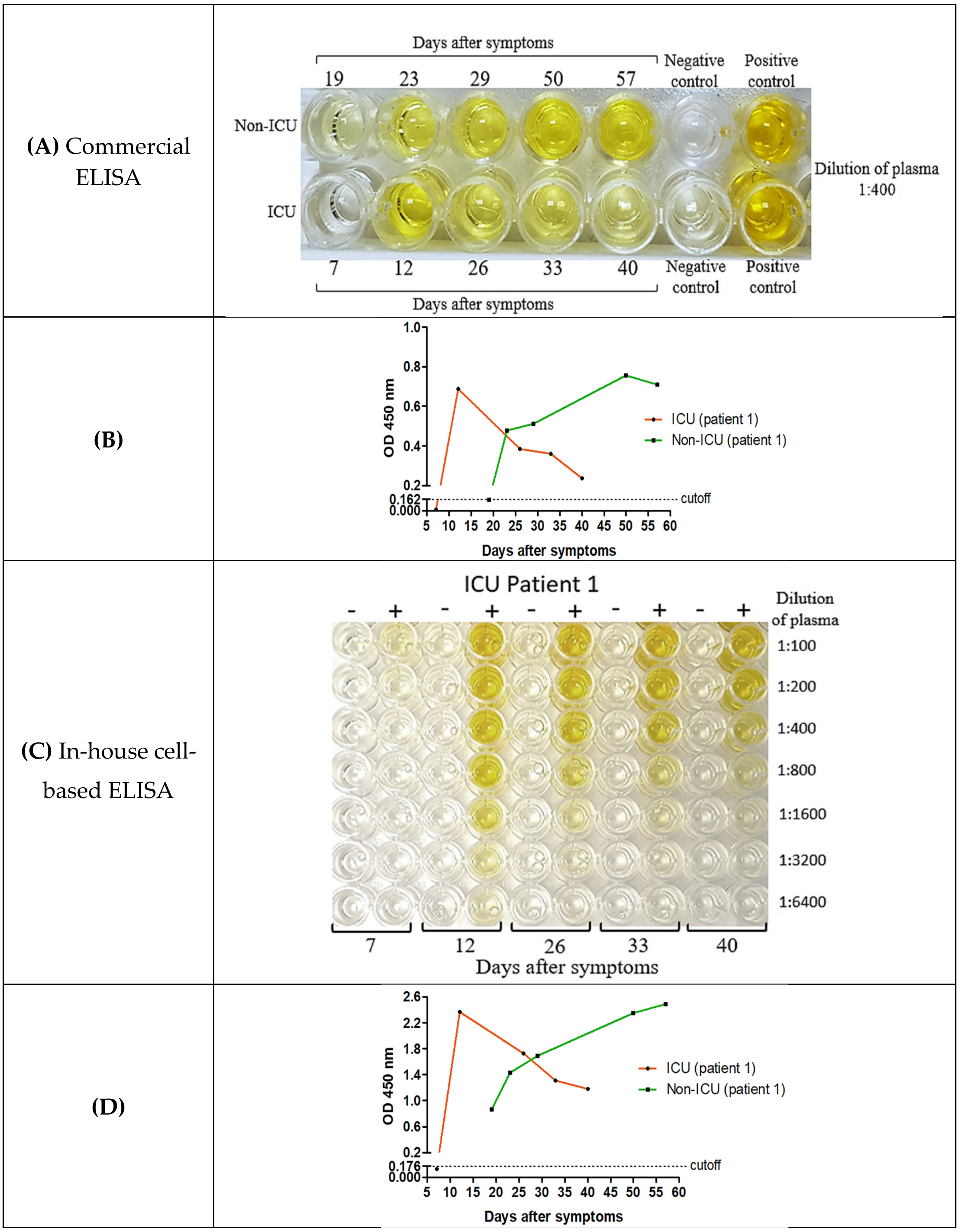

Figure S1. Detection of anti-SARS-CoV-2 antibodies in one ICU and one non-ICU patient, over time, using a commercial and an in-house cell-based ELISA. A) Picture of the commercial ELISA plate after the revelation of anti-N IgG, and B) graph of OD values (450 nm) when plasmas are diluted 400-fold are shown. C) Picture of the in-house cell-based ELISA plate coated with infected (+) or mock-infected (-) Vero-E6 cells, and D) graph of OD values (450 nm) when plasmas are diluted 400-fold are shown.

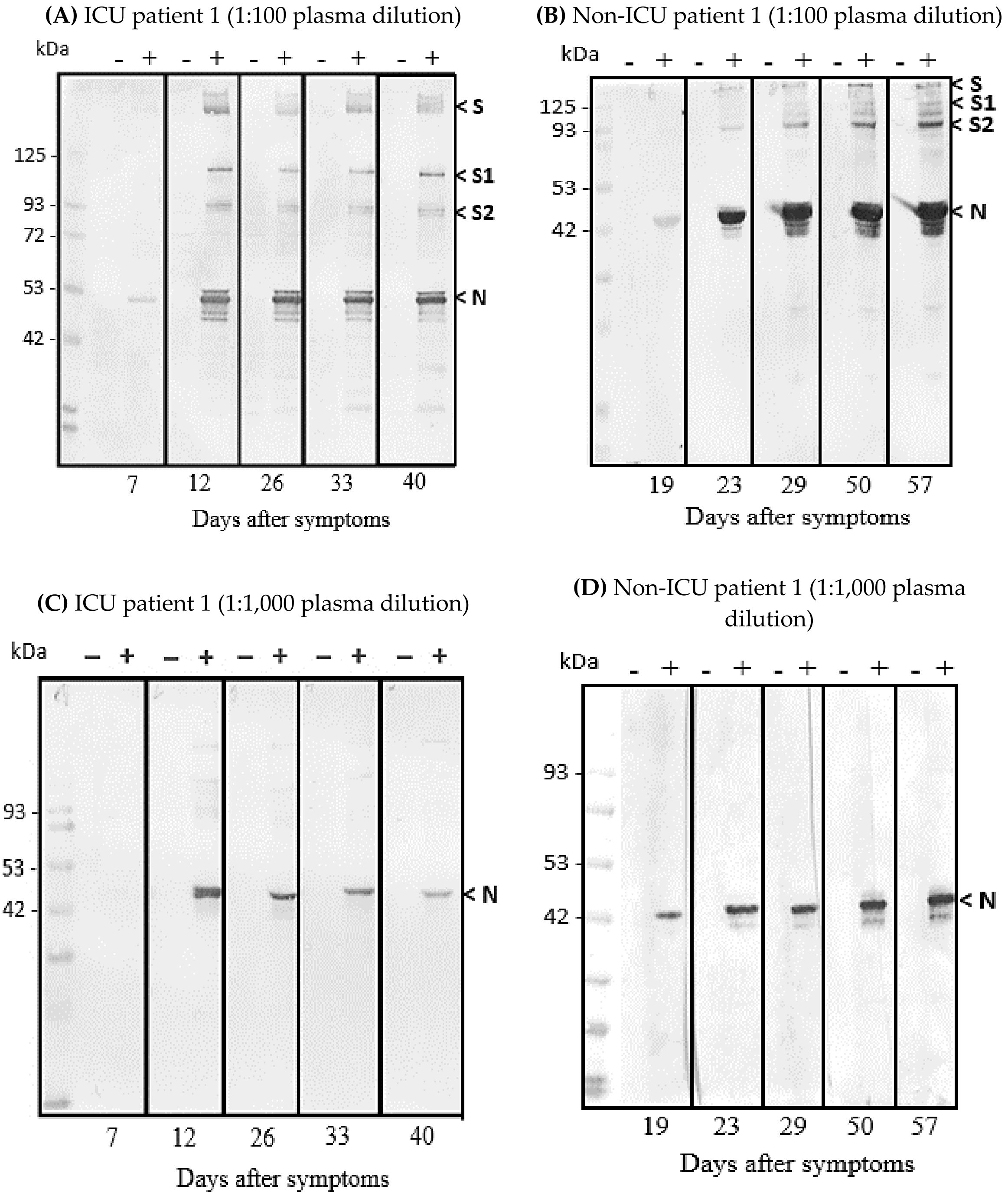

(A) ICU patient 1 (1:100 plasma dilution)
kDa
- + - + - + - + - +
125
93
72
53
42
< S
< S1
< S2
< N
7 12 26 33 40
Days after symptoms
(B) Non-ICU patient 1 (1:100 plasma dilution)
kDa
- + - + - + - + - +
125
93
53
42
< S
< S1
< S2
< N
19 23 29 50 57
Days after symptoms
(C) ICU patient 1 (1:1,000 plasma dilution)
kDa
– + – + – + – + – +
93
53
42
< N
7 12 26 33 40
Days after symptoms
(D) Non-ICU patient 1 (1:1,000 plasma dilution)
kDa
- + - + - + - + - +
93
53
42
< N
19 23 29 50 57
Days after symptoms

(E)

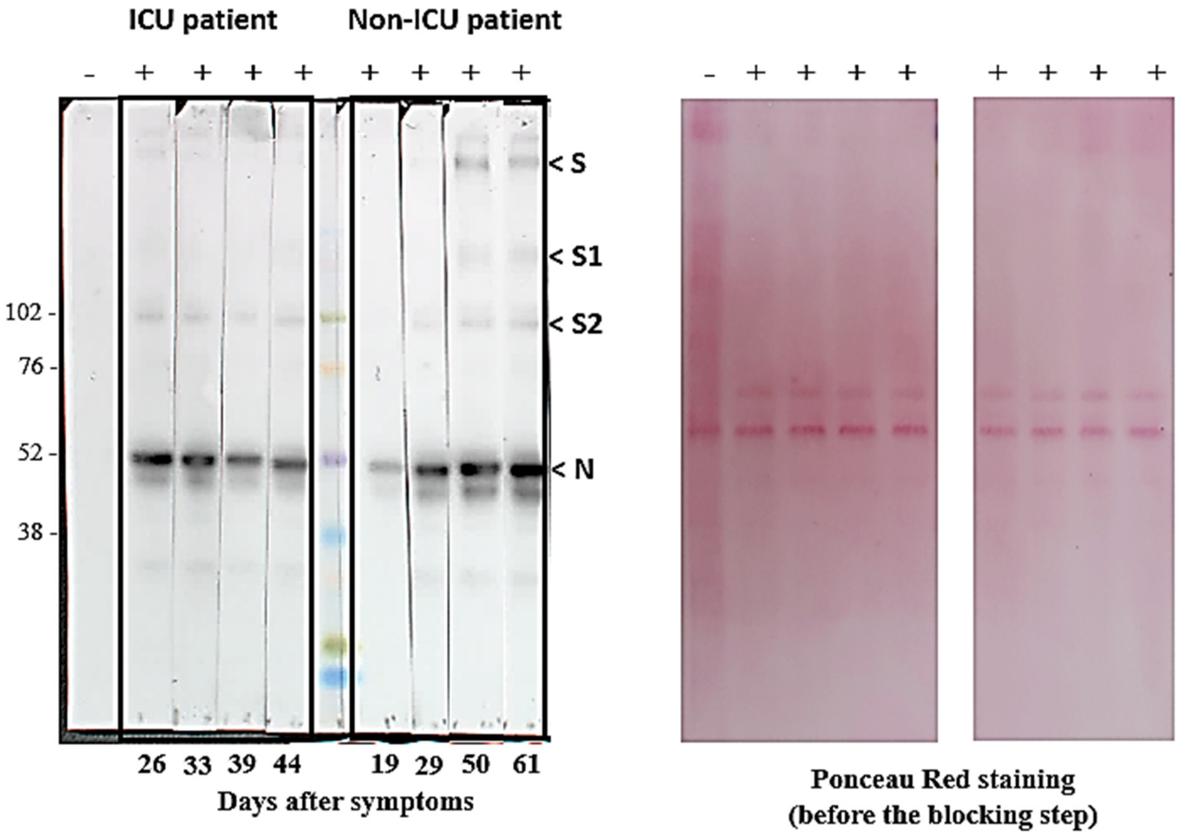

Figure S2. Western blot of mock-infected Vero E6 cells (-) and SARS-CoV-2 infected cells (+) using plasmas of one ICU and one non-ICU hospitalized patient. Samples were diluted either A-B) 100-fold, or C-D) 1,000-fold. Anti-SARS-CoV-2 IgG were revealed using a peroxidase substrate. E) Left panel: Anti-SARS-CoV-2 IgG revelation using enhanced chemiluminescence method. Dilution of plasma samples: 1:1000. (-): mock-infected Vero cell lysate and (+) virus-infected very cell lysates. Right panel: membrane used to perform the WB and stained with Ponceau Red to check for equal total cellular protein loads.
